# Enhancement of Volumetric Capacitance of Binder-Free Single-Walled Carbon Nanotube Film via Fluorination

**DOI:** 10.3390/nano11051135

**Published:** 2021-04-27

**Authors:** Olga A. Gurova, Vitalii I. Sysoev, Egor V. Lobiak, Anna A. Makarova, Igor P. Asanov, Alexander V. Okotrub, Leonid V. Kulik, Lyubov G. Bulusheva

**Affiliations:** 1 Nikolaev Institute of Inorganic Chemistry, Siberian Branch of the Russian Academy of Sciences, 630090 Novosibirsk, Russia; sysoev@niic.nsc.ru (V.I.S.); lobiakEV@niic.sbras.ru (E.V.L.); asan@niic.nsc.ru (I.P.A.); spectrum@niic.nsc.ru (A.V.O.); 2 Physical Chemistry, Institute of Chemistry and Biochemistry, Free University of Berlin, 14195 Berlin, Germany; anna.makarova@helmholtz-berlin.de; 3 V.V. Voevodsky Institute of Chemical Kinetics and Combustion, Siberian Branch of the Russian Academy of Sciences, 630090 Novosibirsk, Russia; chemphy@kinetics.nsc.ru

**Keywords:** single-walled carbon nanotubes, 1,2-dichlorobenzene, fluorination, binder-free film, 1M H_2_SO_4_ electrolyte, electrochemical capacitors

## Abstract

Robust electrode materials without the addition of binders allow increasing efficiency of electrical storage devices. We demonstrate the fabrication of binder-free electrodes from modified single-walled carbon nanotubes (SWCNTs) for electrochemical double-layer capacitors (EDLCs). Modification of SWCNTs included a sonication in 1,2-dichlorobenzene and/or fluorination with gaseous BrF_3_ at room temperature. The sonication caused the shortening of SWCNTs and the splitting of their bundles. As a result, the film prepared from such SWCNTs had a higher density and attached a larger amount of fluorine as compared to the film from non-sonicated SWCNTs. In EDLCs with 1M H_2_SO_4_ electrolyte, the fluorinated films were gradually defluorinated, which lead to an increase of the specific capacitance by 2.5–4 times in comparison with the initial values. Although the highest gravimetric capacitance (29 F g^−1^ at 100 mV s^−1^) was observed for the binder-free film from non-modified SWCNT, the fluorinated film from the sonicated SWCNTs had an enhanced volumetric capacitance (44 F cm^−3^ at 100 mV s^−1^). Initial SWCNT films and defluorinated films showed stable work in EDLCs during several thousand cycles.

## 1. Introduction

The depletion of fossil fuels causes fluctuations in their price and leads to problems with pollution of air and soil. Therefore, recently there has been a growing interest in the development of highly efficient energy storage devices, including solar cells [1,2], rechargeable batteries [3], and electrochemical double-layer capacitors (EDLCs) [4,5]. EDLCs provide high power density, a long-term cycle life, and are environmentally friendly [6]. On the contrary, batteries possess high energy density, but they have low power density [7,8].

The widespread candidates for electrode materials for the EDLC industry are porous carbon in different forms, such as carbon aerogels, soot, activated carbon, and carbon cloth [7,9]. Porous types of carbon materials are widely used in EDLCs, because they have a high specific surface area and developed microporous structure, but they are unfavorable for electrolyte wetting and rapid ionic transport [10]. Moreover, for the fabrication of electrodes from porous carbon materials, a polymer binder is required to hold the carbon particles together. When the mass of the electrochemically inactive binder is taken into account, the specific capacitance of the electrode decreases [11]. 

Carbon nanotubes (CNTs) have a cylindrical structure and possess a high specific surface area, stability, and electrical conductivity, which are factors suggesting that the CNTs are promising materials for polarizable electrodes [9,12]. They are classified as single-walled CNTs (SWCNTs) and multi-walled CNTs (MWCNTs) [13]. In the pure form, CNTs provide moderate values of capacitance (20–60 F g^−1^) [14]. The CNTs have a long and flexible body and hence they are attractive for the fabrication of mechanically stable binder-free films [14,15]. 

To improve the potential application of carbon materials as electrodes in energy storage devices, covalent functionalization (O, halogens, and amines) [9] and heteroatom (N, S, P, and B) doping are widely used [16,17]. The strong electronegativity and the capability of fluorine (F) to form covalent bonds with carbon atoms induce surface polarity that creates more sites for electrolyte ion adsorption, promote ion transportation, and enhance surface wettability [18,19]. For example, Lee et al. demonstrated that the presence of fluorine in the structure of SWCNTs led to their efficient dispersion in water, while the specific capacitance of heat-treated F-SWCNTs increased due to the appearance of micropores [20]. Fluorination of MWCNTs brought similar effects in the increased specific capacitance of the F-MWCNT-based electrode [21]. In addition, there are a few works devoted to the fluorination of CNT buckypapers or nanopapers obtained by filtering CNTs dispersed in a solvent [22,23,24]. In spite of the synthesis, characterization, and different applications of F-CNTs being widely studied, the use of F-CNTs in EDLCs has received little attention. 

Here, we present a study of the effect of fluorination of SWCNT films on their electrochemical behavior when used as binder-free electrodes in 1M H_2_SO_4_ electrolyte. Commercial TUBALL^TM^ nanotubes produced in huge quantities were used. We compare the ability of as-synthesized bundled SWCNTs and individualized SWCNTs to form a compact film and to be fluorinated in mild conditions.

## 2. Materials and Methods

### 2.1. Purification of SWNTs

SWCNTs produced by the OCSiAl company (Novosibirsk, Russia) were purified by a procedure described elsewhere [25]. The ends of the nanotubes were opened by thermal treatment at 500 °C in the air atmosphere. The residual catalyst was removed by sonication of opened SWCNTs in the concentrated HCl for 5 h using a bath. The sample was washed with distilled water until the neutral pH and dried at 100 °C for 2 h in a furnace. The obtained SWCNT powder was dispersed in an aqueous solution of sodium dodecyl sulfate (5 mg mL^−1^) at continuous stirring and the suspension was passed through a silicone capillary placed between permanent magnets for more complete removal of metal nanoparticles. After that, the SWCNTs were washed with distilled water and dried at 100 °C for 2 h in a furnace.

### 2.2. Preparation and Fluorination of Films

To split the bundles, the purified SWCNTs (5 mg) were sonicated using a tip at a power of 100 W in 25 mL of 1,2-dichlorobenzene for 30 min. The suspension was filtrated through a cellulose nitrate (CN) membrane and the precipitate was dried in air. 

A sample (40 mg) of the purified SWCNTs (SW) or the split SWCNTs (SW_DC) was sonicated using a tip in 40 mL of chloroform. The resultant suspensions were vacuum filtered through a CN membrane and the obtained films were dried in air. To remove the residual solvents, the films were annealed in argon at 900 °C for 1 h. 

The SW and SW_DC films were fluorinated using a mixture of BrF_3_ (20 wt.%) and Br_2_ at room temperature. The mixture was poured into Teflon reactor, the SWCNT film was put into a perforated Teflon beaker, the beaker was set on the support in the reactor, which was closed hermetically. After 7 days of the reaction, the samples were purged with N_2_ for 1 day to eliminate residual amounts of unreacted Br_2_ and BrF_3_. The fluorinated CNTs are denoted as F_SW and F_SW_DC.

### 2.3. Characterization Methods

Structures of SWCNTs and films were studied by SEM on a JEOL JSM-6700 F (JEOL Ltd., Tokyo, Japan) microscope and TEM on a JEOL-2010 microscope (JEOL Ltd., Tokyo, Japan). Raman analysis was performed using a LabRAM HR Evolution HORIBA spectrometer (Horiba, Kyoto, Japan) with a 514 nm argon laser.

X-ray photoelectron spectroscopy (XPS) experiments were carried out for initial fluorinated SWCNTs at an excitation of 830 eV of the Russian–German beamline at the Berliner Elektronenspeicherring für Synchrotronstrahlung (BESSY II) station. The binding energies were aligned to the position of the Au 4f_7/2_ line at 84 eV. XPS spectra of the films after electrochemical tests were recorded on a SpecsLab PHOIBOS 150 spectrometer (Specs GmbH, Berlin, Germany) using a monochromatized Al K_a_-radiation (1486 eV). The binding energy scale was calibrated to the energy of 284.5 eV for the sp^2^–carbon component. The analysis of XPS spectra was performed with CASA XPS software, Version 2.3.15 (Casa Software Ltd., Teignmouth, UK) using Gaussian-Lorentzian and Doniach-Sunjic functions after subtraction of the background signal by Shirley’s method.

Nitrogen adsorption/desorption experiments were carried out at liquid nitrogen temperature using a Sorbi MS analyzer (CJSC “META”, Novosibirsk, Russia). The specific surface area (SSA) was calculated by the Brunauer–Emmett–Teller (BET) method based on the data of adsorption isotherms.

### 2.4. Electrochemical Measurements

The binder-free films (0.5 × 0.5 cm^2^) were tested in three-electrode flat cells, assembled using a standard technique [26]. An Ag/AgCl electrode was used as the reference electrode and Pt plates were used as the counter electrode and the current collector for the working electrode. Cyclic voltammetry (CV) curves were recorded in 1M H_2_SO_4_ electrolyte at a scan rate from 2 to 1000 mV s^−1^ on a SP-300 potentiostat/galvanostat (Bio-Logic Science Instrument, Seyssinet-Pariset, France). 

Long-term tests were performed using symmetric two-electrode cells. The gravimetric and volumetric capacitance was calculated from the area of CV curves as: $C_{G}=\frac{\int_{I>0}IdU}{△U\Delta \nu_{s}\cdot m_{el}}\;\mathrm{and}\;C_{V}=\frac{\int_{I>0}IdU}{△U\Delta \nu_{s}\cdot V_{el}}$, respectively, where *I* is the current (A), *dU* is the differentially small increment of potential (V), ∆U is the voltage window (V), $\nu_{s}$ is the scan rate (V s^−1^), $m_{el}$ is the mass of SWCNT film (g), and $V_{el}$. is the volume of the film (cm^3^). The gravimetric capacitance from galvanostatic charge/discharge (GCD) curves was determined as: $C_{G}=\frac{2E_{int/D}}{\Delta U^{2}}$, where $E_{int/D}$ is discharge energy, calculated as $E_{int/D}=I\int_{t\left(U_{max}\right)}^{t\left(U_{min}\right)}U\left(t\right)dt$. 

To study the samples after long-term cycling, the films were washed with a large amount of distilled water and dried in air. 

## 3. Results

### 3.1. Structural Aspects

**Figure 1:**
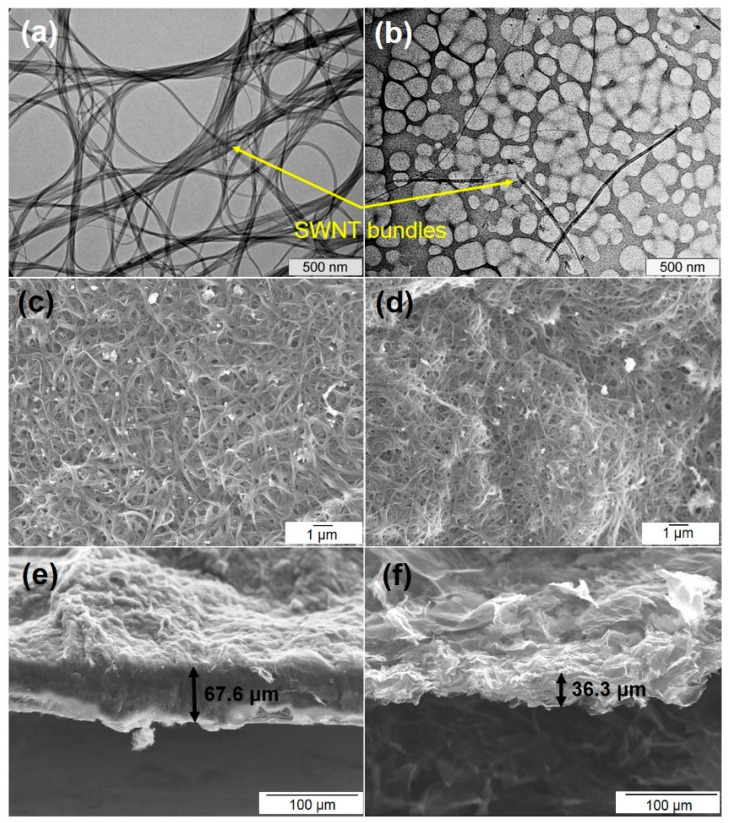
TEM images of purified SWCNTs before (sample SW, (**a**)) and after (sample SW_DC, (**b**)) the sonication in 1,2-dichlorobenzene and SEM images of top-view (**c**,**d**) and side-view (**e**,**f**) of the films prepared from sample SW (**c**,**e**) and sample SW_DC (**d**,**f**).

TEM images of SWCNTs before (sample SW) and after the sonication in 1,2-dichlorobenzene (sample SW_DC) are compared in Figure 1a,b. The former sample consists of entangled SWCNT bundles with an average diameter of ca. 100–200 nm (Figure 1a). The bundles are split and the SWCNTs are shortened substantially in the sample SW_DC (Figure 1b). Filtration of the suspension of short and almost individualized nanotubes results in the formation of a compact and denser film as compared to that produced from the suspension of the SW sample (Figure 1c,d). SEM images of a cross-section of the films reveal that the film SW is 1.9 times thicker than the film SW_DC (Figure 1e,f). The estimated density for the SW and SW_DC films is 0.47 and 0.88 g cm^−3^, respectively.

**Figure 2:**
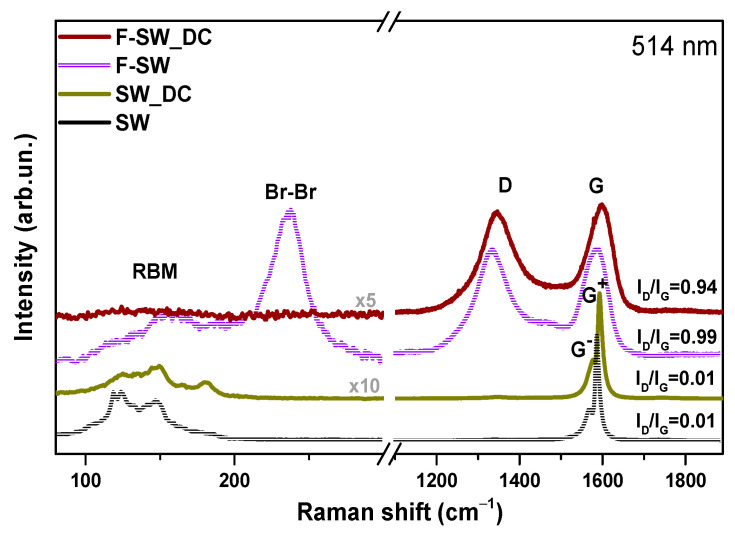
Raman spectra of initial SW and SW_DC films and those after fluorination.

Raman spectra of SW and SW_DC films before and after fluorination recorded in the range of 70 to 2000 cm^−1^ are shown in Figure 2. The spectra of initial SW and SW-DC films exhibit radial breathing modes (RBM) and tangential G band around 1580 cm^–1^ [27]. The appearance of the disorder-induced D band at 1343 cm^−1^ in the Raman spectrum of the SWCNT sample indicates carbon impurities and structural defects in the nanotube walls [28]. Since the TEM study of the SW sample found no impurities (Figure 1a), the ratio of the intensities of D and G bands (I_D_/I_G_) could be used to estimate SWCNT defects. The tangential G band of the SWCNTs splits into G^−^ and G^+^ components located at 1574 and 1592 cm^−1^ and assigned to atomic displacement along the circumferential direction and the tube axis, respectively [29]. The G^−^ component is highly dependent on the laser excitation energy and the geometry (diameter and chiral angle) of the SWCNT [30]. Therefore, we used the peak intensity of the G^+^ component to obtain the I_D_/I_G_ ratio. A very low value of the ratio for the SW film (Figure 2) indicates a high structural order of the studied SWCNTs. The I_D_/I_G_ ratio remains unchanged after the sonication of SWCNTs in a 1,2-dichlorobenzene, meaning that no new defects have arisen in the nanotube walls. However, the Raman spectra of SW and SW_DC films are different in the RBM mode region. The significant variability in relative intensities in this region is due to the inter-tube contact area, which increases with the SWCNT aggregation [29]. Hence, based on the TEM and Raman spectroscopy data, we conclude the main role of the 1,2-dichlorobenzene treatment is the splitting of bundles and cutting of the nanotubes without noticeable functionalization and disordering of their walls. 

The intensity of the D band becomes comparable with the G band intensity in Raman spectra of F-SW and F-SW_DC films. This event and the merging of G^+^ and G^−^ components into one G peak are due to the random creation of covalent C–F bonds that distort the translational nanotube symmetry. Strong suppression of RBMs in the spectrum of F-SW_DC may indicate a high fluorination degree of this sample. The spectrum of F-SW film exhibits an intense band at 236 cm^−1^ assigned to Br_2_ molecules intercalated between nanotubes [31] The absence of this band in the spectrum of F-SW_DC film is due to the splitting of the bundles.

XPS study reveals the difference between F-SW and F-SW_DC films in the degree of fluorination. According to the XPS survey spectra (not shown), the fluorinated films contain carbon and fluorine as the main elements and a small amount of bromine and oxygen. The fluorine content in F-SW and F-SW_DC is about 14 and 23 at%, respectively. 

**Figure 3:**
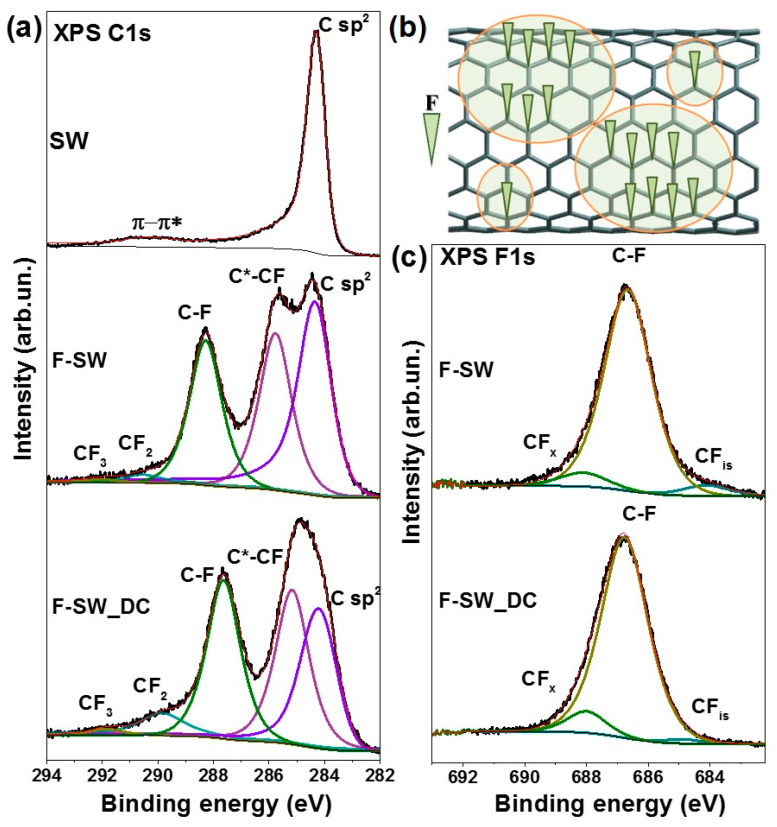
XPS C 1s spectra of initial SW film and fluorinated F-SW and F-SW_DC films (**a**). Schematic presentation of fluorine distribution on the SWCNT surface (**b**). XPS F 1s spectra of F-SW and F-SW_DC films (**c**).

XPS C 1s spectra of SW, F-SW, and F-SW_DC films are compared in Figure 3a. The spectrum of purified SWCNTs is presented by an asymmetric line at 284.3 eV corresponding to the sp^2^-hybridized carbon and a broad high-energy line assigned to π-π* satellites [32]. XPS C 1s spectra of the fluorinated films contain five components associated with different chemical states of carbon. Both spectra have the C sp^2^ component and this indicates that fluorine-free areas remain on the nanotube surface. The fraction of such areas is larger in the F-SW sample. There, the SWCNTs are gathered into big bundles. The surface of the bundle is fluorinated first and this limits penetration of the fluorinating agent to the interior nanotubes. The splitting of the bundles in SW_DC makes most of the nanotubes available for the fluorination and as a result, the sample is more uniformly fluorinated. As compared to non-fluorinated SWCNTs, the C sp^2^ component is upshifted by 0.1 eV for F-SW and by 0.2 eV for F-SW_DC. This upshift is due to the transfer of electron density from carbon to the fluorine atom. The C 1s spectra of F-SW and F-SW_DC films also show two intense components at 285.7 and 288.4 eV attributed to the carbon neighboring with CF-group (C*-CF) and the carbon covalently bonded with fluorine (C-F), respectively [33,34]. The components at 290.5 and 292.5 eV correspond to the edge CF_2_ and CF_3_ groups, respectively [35]. The fraction of CF_2_ groups is larger in the F-SW-DC sample due to the shortening of the nanotubes as a result of their sonication in 1,2-dichlorobenzene. 

Based on the analysis of the XPS C 1s spectra, we conclude that the distribution of fluorine on the SWCNT surface is uneven. An intense C sp^2^ component, observed in the spectra of F-SW and F-SW_DC samples (Figure 3a), provides evidence of preservation of the conjugated π-electron areas. Therefore, the nanotube surface is a composition of sp^2^-carbon areas and CF_x_ domains (Figure 3b). The average fluorine content x in the CF_x_ domains is determined using the areas of the C 1s spectrum components as x = I_C-F_/(I_C-F_ + I_C*-CF_). This value is 0.4 in F-SW_DC and 0.48 in F-SW. Previously, it has been shown that the preferred pattern in the layers of fluorinated graphite of the CF_0.5_ composition is the alternate chains from CF groups and bare carbon atoms [36]. Since the C*-CF/C-F ratio is larger in the spectrum of the F-SW_DC sample, we conclude the CF chains are shorter in the CF_x_ domains for these nanotubes. In addition, some fluorine atoms are isolated, i.e., they have no CF neighbors. The fraction of such fluorine atoms is larger in the F-SW sample, where the XPS F 1s spectrum clearly shows the low-energy component CF_is_ (Figure 3c). However, most of the fluorine atoms form covalent bonds with the SWCNT surface that is evident from a dominant line at 686.7 eV [19,37] in the spectra of both fluorinated samples. A high-energy component at 688.1 eV is attributed to fluorine from edge CF_x_ groups [38]. The intensity of this component is higher in the spectrum of F-SW_DC in accordance with the C 1s spectral data (Figure 3a). 

### 3.2. Electrochemical Behavior of SWCNT-Based Electrodes

The operational voltage of carbon capacitors with aqueous electrolytes is usually below 1 V [39] and this value is limited by the electrochemical stability of water (thermodynamically 1.23 V) [40] and also controlled by the electrode material and the electrolyte pH [41]. The tests of our materials showed that the optimal window in 1M H_2_SO_4_ electrolyte is between 0 and 0.9 V. At these potentials, irreversible processes in the cells are insignificant. Electrochemical performances of SWCNT films are evaluated using CV measurements in two-electrode cells.

**Figure 4:**
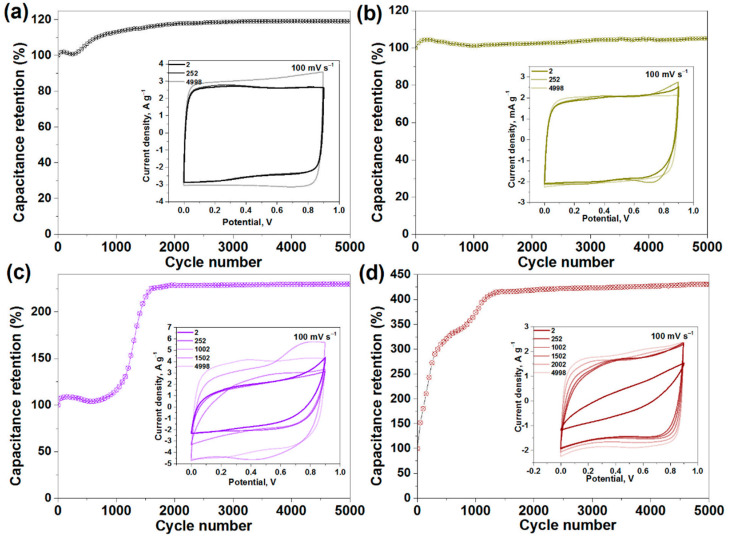
Capacitance retention for SW film (**a**), SW_DC film (**b**), F-SW film (**c**) and F-SW_DC film (**d**) during long-term cycling in 1M H_2_SO_4_ electrolyte at a scan rate of 100 mV s^−1^ in two-electrode cells. Insets show the CV curves measured at 2nd, 252nd, 1002th, 1502th, and 4998th cycles.

The data on long-term cycling are presented in Figure 4. The CV curves recorded for the non-fluorinated SW and SW_DC electrodes at a scan rate of 100 mV s^−1^ show a nearly rectangular shape characteristic for EDLCs (inserts in Figure 4a,b). The CV loops slightly increase with long-term cycling owing to the impregnation of electrodes with 1M H_2_SO_4_ electrolyte [42]. This process is more substantial for the SW film and it is accompanied by the specific capacitance growth by 20% during the long-term cycling (Figure 4a). In contrast, the capacitance of the cell with the SW_DC electrodes is constant (Figure 4b). The main differences between SW and SW_DC are the density of the films and the size of the nanotube bundles. Although the SW_DC film is denser, this does not hinder the diffusion of electrolyte ions into the depth of the film. The surface of well-split nanotubes in this sample is easily accessible for the ions. However, this not the case for the SW film, where the SWCNTs form big-size bundles. It takes time for electrolyte ions to penetrate to the SWCNTs in the center of the bundle. 

CV curves of the fluorinated films exhibit a slope at the first cycles (insets in Figure 4c,d), which is especially marked for the F-SW_DC film. The shape of the curves changes with the cycling, gradually approaching a rectangular shape. Since such shape characterizes a double-layer capacitive behavior of pure carbon materials [43], we conclude electrochemical defluorination of F-SW and F-SW_DC films. An increase in the capacitance retention of the cells (Figure 4c,d) indicates the lower specific capacitance of the starting fluorinated SWCNTs. The specific capacitance increases by about 2.5 times for F-SW and 4 times for F-SW_DC after 2000 cycles and keeps unchanged during further cycling. The results on the long-term cycling of the studied samples generally show the good stability of binder-free SWCNT films.

**Figure 5:**
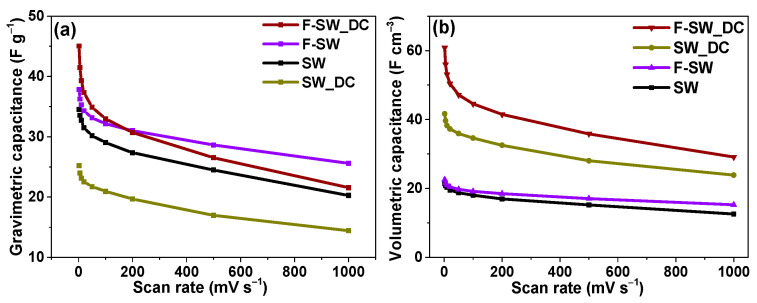
Gravimetric capacitance (**a**) and volumetric capacitance (**b**) of binder-free SWCNT-based films depending on scan rate. The measurements were performed in three-electrode cells.

After long-term tests, the films were studied at various scan rates. When calculating the gravimetric capacity of fluorinated films, we used the weight of the electrodes taken out of the cells, washed with distilled water, and dried in air. Because of electrochemical defluorination, films F-SW and F-SW_DC lost ~37% and ~38% of the initial weight, respectively. Dependences on the gravimetric capacitance on scan rates show better performances for F-SW and F-SW_DC as compared with the non-fluorinated parent samples (Figure 5a). The F-SW_DC film has larger capacitance at scan rates up to 200 mV s^−1^ than the F-SW film. The dense SW_DC film has the smallest values of specific capacitance. However, the films composed of split SWCNTs significantly exceed the bundled samples SW and SW_DC in volumetric capacitance (Figure 5b). The largest value is achieved for the F-SW_DC film. Thus, the splitting of SWCNTs bundles with the following fluorination is a useful way to obtain dense electrodes with an advanced volumetric capacitance. 

### 3.3. F-SWCNTs after Electrochemical Tests

**Figure 6:**
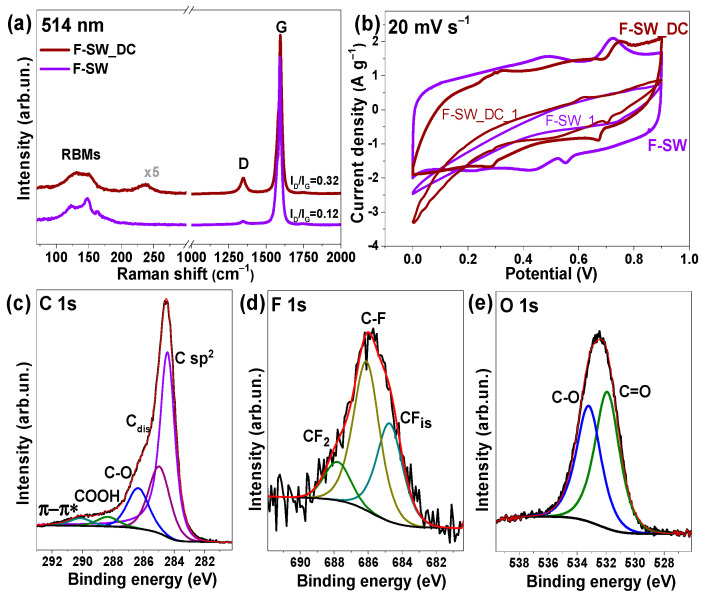
Raman spectra of F-SW and F-SW_DC films after long-term cycling (**a**). CV curves measured at a scan rate of 20 mV s^−1^ vs. Ag/AgCl electrode for the starting films (F-SW_1 and F-SW_DC_1) and those after long-tern cycling (**b**). XPS C 1s (**c**), F 1s (**d**), and O 1s (**e**) spectra of the cycled F-SW film.

Structural changes occurring with the fluorinated SWCNT films after long-term cycling were examined by Raman spectroscopy (Figure 6a). As compared to the spectra of initial fluorinated films (Figure 2), the spectra of the cycled films show a dramatic decrease in the intensity of the D band and the width of the G band as well as repairing of RBMs. These spectral changes confirm electrochemical defluorination of the nanotubes.

Figure 6b compares the CV curves measured at the first cycle and after the long-term cycling of F-SW and F-SW_DC in three-electrode cells. The initial CV curves have a shuttle shape that indicates a low current density and poor conductivity of the films [44] due to a decreased amount of π-electrons after fluorination. Long-term cycling leads to the change in the CV curves to more rectangular-like shapes. Two pairs of redox peaks are clearly visible on the curves. The pair at 0.48/0.44 V in the CV curve of F-SW and 0.33/0.28 V in the CV curve of F-SW_DC corresponds to oxygen-containing groups and carbonyl and quinone oxygen groups are electrochemically active in an acidic electrolyte [45,46]. The pair at 0.72–0.75/0.56–0.66 V could be assigned to fluorine contribution since the CV curves of the starting fluorinated films have peaks at close values of the potential.

The XPS study of electrochemically treated F-SW film detected a decrease in the fluorine content of less than 1 at% and an increase in the oxygen content of ~11 at% versus ~3 at% in the initial samples. The C 1s spectrum of the cycled F-SW electrode contains no components from carbon bonded with fluorine (Figure 6c). The components at ~286.4 and ~288.5 eV correspond to the carbon bonded with oxygen in hydroxyl and carboxyl groups, respectively [47]. A high component C_dis_ at ~285.0 eV is assigned to the sp^2^-carbon atoms located near the oxygen functionalities as well as the disordered states [48,49] formed in the SWCNT walls after removal of fluorine.

The XPS F 1s spectrum of the cycled F-SW electrode detects a decrease of the amount of covalent CF bonds as compared to the edged CF_2_ groups and the isolated CF groups (Figure 6d). The O 1s spectrum exhibits two components located at ~531.9 and ~533.1 eV (Figure 6e) from oxygen correspondently doubly and singly bound to carbon [50].

### 3.4. Electrochemical Performance of Aged Electrodes

Results presented in Figure 4 show that electrochemical behavior of the SWCNT-based films changes with the cycling. An increase in the specific capacitance of non-fluorinated SW and SW_DC samples is due to wetting of electrode with electrolyte. Strong changes detected for the fluorinated films F-SW and F-SW_DC are related to a defluorination process occurred in an electrochemical cell. 

**Figure 7:**
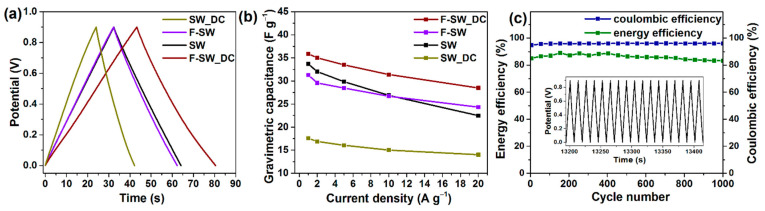
GCD curves of SWCNT-based films at the current density of 1 A g^−1^ (**a**). Dependencies of gravimetric capacitance on current density (**b**). Energy and coulombic efficiencies of F-SW_DC electrode at 5 A g^−1^ (**c**). Inset shows the GCD curves for last 17 cycles.

To study the stability of binder-free SWCNT-based electrodes, they were tested again after aging in laboratory air for 1 month. After washing with distilled water, the films were impregnated with a fresh electrolyte and used to assemble two-electrode cells. The measurements were conducted in a galvanostatic regime. Figure 7a compares the GCD curves recorded at a current density of 1 A g^−1^. All electrodes exhibit a symmetrical triangular shape characteristic for a capacitive response when charges fast propagate through the electrode. Specific capacitances calculated from the GCD curves are presented in Figure 7b. At current densities up to 10 A g^−1^ the capacitance increases in the set of the samples SW_DC < F-SW < SW < F-SW_DC. At larger current densities, the trend is the same as that obtained from the CV curves measured at the scan rates less than 200 mV s^−1^ (Figure 5a).

The best F-SW_DC electrode was used to evaluate the electrochemical efficiency of binder-free SWCNT-based films. Energy and coulombic efficiencies are calculated from potential–time dependences obtained at a current density of 5 A g^−1^ (Figure 7c). The energy efficiency is estimated as the ratio of discharge area to charge area of a GCD curve and the coulombic efficiency is equal to the ratio of discharge and charge intervals [51]. Coulombic efficiency of ~96% and a good repeatability of the GCD curves for the last 17 cycles (inset in Figure 7c) demonstrate high reversibility of F-SW_DC electrode after long-term cycling tests. The energy efficiency of the electrode varies from 84 to 88% (Figure 7c). Reduced energy efficiency can be due to irreversible processes such as the loss of redox-active groups, decomposition of the electrolyte, and the block of the pores in the electrode material. 

## 4. Discussion

The fabrication of robust electrode materials without the addition of binders allows increasing efficiency of electrical storage devices [52,53]. CNTs are attractive for this purpose because one-dimensional and flexible structures are easily intertwined to form a mechanically stable film [52]. Such films can be produced directly in the synthesis [54] or prepared by filtration of a suspension containing CNTs [55].

EDLCs store energy via the adsorption of electrolyte ions at the electrode material surface [56]. Hence, a large surface area is a necessary condition for the high specific capacitance of the device. Among the materials studied, SW possesses the largest specific surface area, while it shows average gravimetric capacitance (Table 1). In this work, we use commercial SWCNTs with the good atomic ordering of the walls, which are produced in quantities sufficient for practical use. These SWCNTs are gathered in long, large-diameter bundles that are difficult to disperse. However, the sonication of the SWCNTs in 1,2-dichlorobenzene splits the bundles and shortens the nanotubes [57]. We show that the filtration of aqueous suspensions containing equal weights of the initial SWCNTs and the split SWCNTs yields films of significantly different thicknesses (Figure 1e,f). The density of the film increases and the specific surface area decreases after the SWCNT splitting (SW_DC sample). The reason is probably the strong adhesion of the shortened SWCNTs. In comparison with the initial SW film, the SW_DC film has lower values of gravimetric capacitance while it shows a larger volumetric capacitance (Table 1). Moreover, its CV loop practically did not change during the EDLC operation for about 5000 cycles (inset in Figure 4b), which indicates the high stability of the SW_DC film and its advantage for practical use.

Fluorination is considered a way to improve the performance of carbon materials in EDLCs [9]. The reason for this improvement is usually cited as fluorine-induced surface polarization resulting in the adsorption of more electrolyte ions. Note, that there are very few studies on the electrochemical behavior of the fluorinated carbon in aqueous electrolytes and they concern MWCNTs [58], carbon microspheres [59], activated carbon [60], and graphitic materials [61]. We are not aware of any work on the investigation of the fluorinated SWCNTs in the EDLCs.

To fluorinate the SW and SW_DC films, we used a mixture of BrF_3_ and Br_2_ vapors at room temperature. Earlier, we showed that this method does not destroy the tubular structure while in the case of the assembled SWCNTs, mainly the surface of the bundles is fluorinated [62]. This result was confirmed in the present study by XPS and N_2_ adsorption measurements. The XPS examination found a larger fluorine content in the F-SW_DC film enriched with the individualized nanotubes. The specific surface area of SW and SW_DC films decreased after the fluorination by a factor of 1.5 and 1.7 (Table 1), respectively, and this result indicates the higher population of the SWCNT surface with fluorine in the latter case.

A part of the carbon in the SWCNT walls remains non-fluorinated and this provides electrical conductivity of the samples necessary for the operation of the EDLC. The initial CV curve of highly fluorinated SW-DC film has a small loop and a large slope (inset in Figure 4d) because of the low conductivity of the electrode. The specific capacitance of the fluorinated films grew during the first 1300–1500 cycles of the EDLC work and then it stabilized (Figure 4c,d). The values of the capacitance reached 32–33 F g^−1^ at 100 mV s^−1^ for these films (Table 1).

A study of the films after long-term cycling reveals their defluorination accompanying by the emergence of new oxygen-containing groups, mainly hydroxyl and carboxyl. The carboxyl groups can be attached only to the nanotube edges. Hence, the electrochemical reduction of the fluorinated SWCNTs produces atomic vacancies in the nanotube walls. The breaking of the C=C bonds during the electrochemical treatment was previously observed for MWCNTs in aqueous electrolytes [63]. In the case of the fluorinated SWCNTs, this process may be easier due to a high tension of (F)C–C(F) bonds. We expect the outer SWCNT surface to be fluorinated first. Fluorine atoms are preferably attached to the (1,2) positions of carbon hexagons [64] forming the CF chains (Figure 3b). Because of one-side fluorination, the C–C bonds are elongated and consequently, they can break when a potential is applied. Oxygen-containing groups present in the electrolyte terminate the formed dangling bonds. 

The redox-active groups contribute to the capacitance at low scan rates, while at high scan rates the capacitance is dependent on the number of the adsorption sites. The drop in the capacitance with a scan rate was similar for non-functionalized SW and SW_DC samples and it increased significantly for F-SW_DC (Table 1). An optimal balance between the attached surface groups and the area available for the electrolyte leads to a high capacitance retention with the increasing scan rate for the F-SW electrode. Moreover, fluorination increased the volumetric capacitance of the SWCNT-based electrodes (Figure 5b, Table 1). Superior improvement was achieved for the SW_DC sample, which was able to attach more fluorine atoms. The electrochemical detachment of fluorine atoms creates many vacancy defects in the walls of SWCNTs composing the F-SW_DC sample. Large vacancy defects can open access of the solvated electrolyte ions to the interior of the nanotubes and we believe this is the main reason for the growth of the volumetric capacitance at high scan rates of the applied potential. The detached fluorine atoms insignificantly alter electrolyte, because, after its replacement by a fresh portion, the capacitance of the cells remains approximately the same. In an acidic electrolyte, fluorine interacts with a proton to form HF. Considering the fluorine loss and the volume of the electrolyte in the cell, the concentration of HF in the solution is below 0.1 M. 

The volumetric capacitance of the F-SW_DC electrode calculated at 100 mV s^−1^ was 44 F cm^−3^ (Table 1). This value exceeded the volumetric capacitance of activated carbon electrodes (~40 F cm^−3^ [65]), CNTs mixed with carboxymethylcellulose (8–16 F cm^−3^ [65]), and binder-free CNT-based electrodes prepared by the electrostatic spray deposition (2.9 F cm^−3^ [66]) and spray deposition (~42 F cm^−3^ [67]). The largest reported values of the volumetric capacity of CNT-based electrodes are about 44–74 F cm^−3^ and they were obtained in 2M H_2_SO_4_ at 5 mV s^−1^ [68]. Our best electrode delivered 56 F cm^−3^ at this scan rate (Figure 5b).

## 5. Conclusions

In the present paper, the effect of splitting and fluorination of SWCNTs on their electrochemical behavior in 1M H_2_SO_4_ electrolyte was investigated. The sonication of SWCNTs in 1,2-dichlorobenzene was used to split the bundles and to shorten the nanotubes. Mechanically robust films for binder-free electrodes were prepared by vacuum filtration of aqueous suspensions of the bundled SWCNTs (SW) and split SWCNTs (SW_DC). The smaller specific surface area of the latter film was attributed to the stronger adhesion between the shortened nanotubes. Fluorination of the films was carried out in identical conditions using a gaseous mixture of BrF_3_ and Br_2_ at room temperature. According to the XPS survey spectra, the F-SW and F-SW_DC films contained 14 and 23 at% of fluorine, respectively. As a result of prolonged electrochemical exposure at potentials between 0 and 0.9 V, most of the fluorine atoms were removed from the SWCNT walls. Defluorination was accompanied by the creation of atomic vacancies and attachment of oxygen-containing groups. This modification caused enhancement of volumetric capacitance of split and short SWCNTs as compared to other tested films. The film from the bundled SWCNTs (SW) showed superior gravimetric capacitance. Initial SWCNT films and defluorinated films showed a stable work of EDLCs during the several thousand cycles. We tested commercial SWCNTs, produced by OCSiAl company on a large scale, so, our work can open new perspectives for the fabrication of flexible and binder-free electrodes with advanced electrochemical performance.

## Figures and Tables

**Table 1:** Characteristics of studied films: specific surface area (SSA), fluorine content (F), gravimetric capacitance (C_G_) and volumetric capacitance (C_V_) obtained at 100 mV s^−1^, and drop in the gravimetric capacitance with an increase of the scan rate from 2 to 1000 mV s^−1^.

Film	SSA, m^2^ g^−1^	F, at%	C_G_, F g^−1^	C_V_, F cm^−3^	C_G_ Drop, %
SW	1135	–	29	18	41
SW_DC	840	–	21	34	43
F-SW	753	14	32	19	32
F-SW_DC	494	23	33	44	52
